# Six years of fruit fly surveys in Bangladesh: a new species, 33 new country records and discovery of the highly invasive *Bactrocera carambolae* (Diptera, Tephritidae)

**DOI:** 10.3897/zookeys.876.38096

**Published:** 2019-09-25

**Authors:** Luc Leblanc, M. Aftab Hossain, Camiel Doorenweerd, Shakil Ahmed Khan, Mahfuza Momen, Michael San Jose, Daniel Rubinoff

**Affiliations:** 1 University of Idaho, Department of Entomology, Plant Pathology and Nematology (EPPN), 875 Perimeter Drive MS 2329, Moscow, Idaho, USA University of Idaho Moscow United States of America; 2 Insect Biotechnology Division, Institute of Food and Radiation Biology, Bangladesh Atomic Energy Commission, Dhaka-1349, Bangladesh Bangladesh Atomic Energy Commission Dhaka Bangladesh; 3 University of Hawaii at Manoa, Department of Plant and Environmental Protection Sciences, 3050 Maile Way, Gilmore 310, Honolulu, HI 96822, USA University of Hawaii at Manoa Honolulu United States of America

**Keywords:** Dacini, Indian subcontinent, pest species, range extension, taxonomy

## Abstract

We engaged in six years of snap-shot surveys for fruit flies in rural environments and ten protected forest areas of Bangladesh, using traps baited with male lures (cue-lure, methyl eugenol, zingerone). Our work has increased the recorded number of species of Tephritidae in the country from seven to 37. We summarize these surveys and report eight new country occurrence records, and a new species (*Zeugodacus madhupuri* Leblanc & Doorenweerd, **sp. nov.**) is described. The highlight among the new records is the discovery, and significant westward range extension, of *Bactrocera carambolae* Drew & Hancock, a major fruit pest detected in the Chattogram and Sylhet Divisions. We rectify the previously published erroneous record of *Bactrocera bogorensis* (Hardy), which was based on a misidentification of *Zeugodacus diaphorus* (Hendel). We also report the occurrence in Bangladesh of nine other Tephritidae, the rearing of three primary fruit fly parasitoids from *Zeugodacus*, and records of non-target attraction to fruit fly lures.

## Introduction

**Figure 1.**
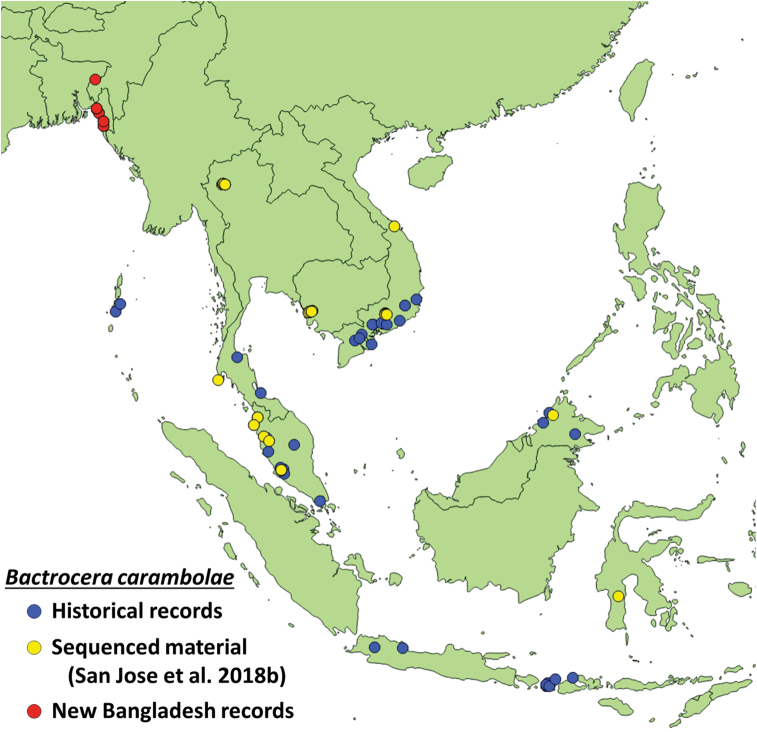
Distribution of *Bactrocera dorsalis* and *B. carambolae* in Asia, including the new records of *B. carambolae* in Bangladesh and range expansion recorded in San Jose et al. (2018b).

The Dacini is a very diverse group of fruit flies, with 939 described species, including 83 pests of cultivated fruit and cucurbits (e.g., Doorenweerd et al. 2018). Of these, 118 are known to occur on the Indian subcontinent (David and Ramani 2011; Drew and Romig 2013; David et al. 2016, 2017; Leblanc et al. 2018b). Fruit fly surveys in rural environments of Bangladesh, initiated in 2013, increased the published number of known species from seven to 27 (Leblanc et al. 2013, 2014; Hossain and Khan 2013; Khan et al. 2015, 2017). While Drew and Romig (2013) could not confirm the presence of *Bactrocera dorsalis* (Hendel) on the Indian subcontinent, variation in color pattern and preliminary molecular data from Bangladesh and African populations suggested that *B. dorsalis* is widespread on the subcontinent and that the species described as *B. invadens* Drew, Tsuruta & White is conspecific with *B. dorsalis* (Leblanc et al. 2013). That same year, *B. philippinensis* Drew & Hancock was declared a synonym of *B. papayae* Drew & Hancock (Drew and Romig 2013). Soon after *B. papayae*, along with *B. invadens*, were declared conspecific with *B. dorsalis*, with formal designation of synonyms (Schutze et al. 2015a, 2015b), leaving *B. carambolae* Drew & Hancock as a distinct species, based on genetic differences, morphological differences in aedeagus, wing shape and color pattern, non-random assortative mating with *B. dorsalis*, and significant differences in pheromone composition (Wee and Tan 2007; Schutze et al. 2012, 2013, 2015b; Tan et al. 2013). With this revised status, *B. dorsalis* is now widespread across tropical Asia, and introduced to most of Africa and several islands in the Pacific, while *B. carambolae* has been restricted to a smaller range in South-East Asia (Fig. 1) and introduced to South America. To generate a complete inventory of the economic species and assess the diversity of fruit flies in the protected forest areas of Bangladesh, we surveyed for fruit flies during 2013–2018 with a focus on rural areas and report here cumulative results from these surveys, focusing on previously unpublished new records. Using a morphological and molecular approach, we discovered numerous new country records, including the highly invasive *B. carambolae*, and a new species of *Zeugodacus* Hendel is described here.

## Material and methods

### Collecting and curation

**Figure 2.**
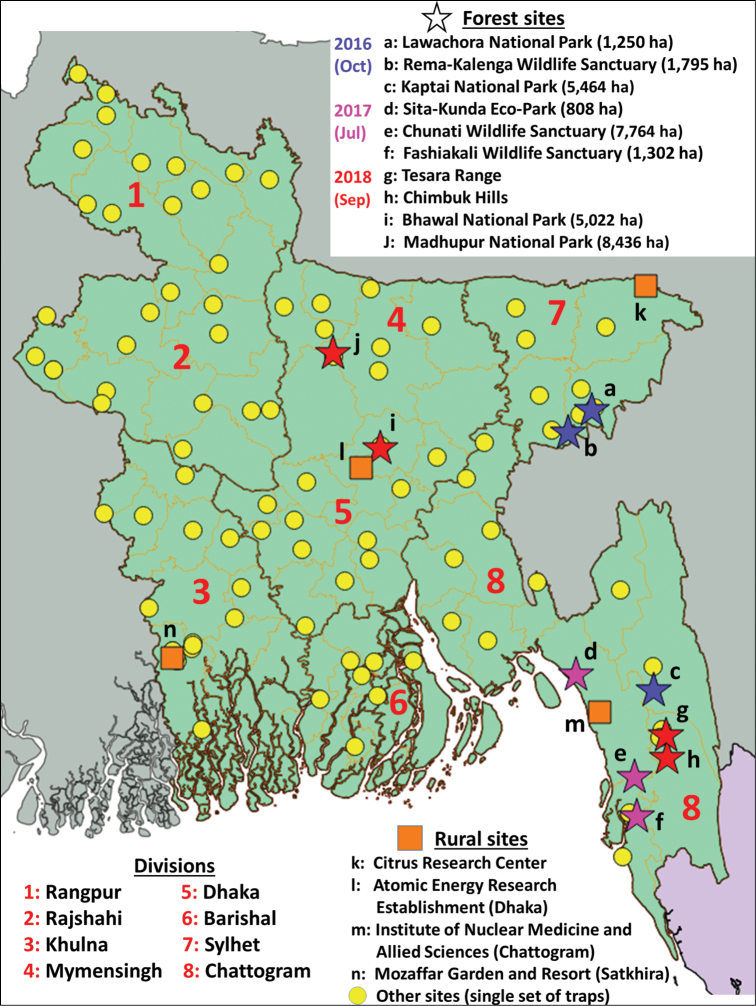
Trapping locations in the various Bangladesh surveys (2013–2018).

Starting in 2013, we periodically maintained a series of traps (described in Leblanc et al. 2015a) separately baited with male lures plus a 10×10 mm piece of dichlorvos (DVVP) insecticide strip to kill trapped flies. Cue-lure and methyl eugenol were included as commercially available plugs (Scentry Biologicals, Billings, Montana) whereas zingerone lure, also used in the surveys since 2016, was prepared by dipping dental cotton wicks in zingerone (= vanillylacetone) (Sigma-Aldrich) melted over a hot plate and allowed to solidify in the wicks. We deployed traps at 383 sites throughout the country for periods ranging from one to 14 days, either as individual sites scattered over rural areas or as series of 11–26 sites, about 50 m apart, concentrated in selected rural areas and in 10 different protected forest areas (Nishorgo Support Project 2007) (Fig. 2, Table 1). Sampled flies were stored in 95% ethanol in a -20 °C freezer, to preserve DNA for analysis. All flies were identified by the first three authors, using available keys (Drew and Romig 2013, 2016). Before drying flies for double-mounting, we pinned them through the scutum with a minuten pin and soaked them in ethyl-ether for 3–12 hours to fix and preserve their natural coloration. We photographed specimens using a Nikon D7100 camera attached to an Olympus SZX10 microscope and used Helicon Focus Pro ver. 6.7.1 to merge pictures taken at a range of focal planes. To measure specimens, we used an ocular grid mounted on an Olympus SZ30 dissecting microscope.

We also reared parasitoids and hyperparasitoids from readily available, heavily fly-infested snake gourd (*Trichosanthes cucumerina*) collected at the AERE campus (Dhaka). Infested gourds were weighed and placed on a cloth-covered small bowl (to collect excess juice from decay), over moist sawdust (as a pupation media) in a fine nylon netted cage. Pupae were separated from the sawdust using fine-meshed sieve and placed in a petri dish inside a very fine-netted plastic cage to collect emerged fruit flies and parasitoids.

**Table 1.** Checklist and distribution of Dacine fruit flies of Bangladesh, including previously known species, new country occurrence records, and number of specimens collected in the surveys (2013–2018) reported in this paper.

Taxa	Male Lure	Barishal	Chattogram							Dhaka			Khulna	Mymensingh	Rajshahi	Rangpur	Sylhet		
		Rural sites (6)	Chimbuk Hills (25)	Chunati (26)	Fashiakali (15)	Kaptai (24)	Sita-Kunda (23)	Tesara Range (25)	Rural sites (46)	Bhawal (15)	Madhupur (15)	Rural sites (49)	Rural sites (25)	Rural sites (7)	Rural sites (16)	Rural sites (13)	Lawachora (22)	Rema-Kalenga (11)	Rural sites (20)
*** Bactrocera ***																			
*B. abbreviata* (Hardy)*	ZN								1										
*B. bhutaniae* Drew & Romig^7^	CL		5																4
*B. carambolae* (Drew & Hancock)*^1^	ME^5^			38	95		25		8									1	
*B. correcta* (Bezzi)^1^	ME				1				563			3							
*B. digressa* Radhakrishnan	CL, ZN									3		1			10				
*B. dorsalis* (Hendel)^1, 5^	ME^5^	19	219	552	548	150	172	216	2811	516	567	2373	825	375	2414	684	50	56	330
*B. latifrons* (Hendel)^2^	Latilure^6^											11							
*B. nigrifacia* Zhang, Chi & Chen	CL	1	1			3	1					28	9	3	2	16			3
*B. nigrofemoralis* White & Tsuruta	CL									1									
*B. pendleburyi* (Perkins)*	ZN			2	3		3												
*B. propinqua* (Hardy & Adachi)	CL		3	6	5	2	6	4		5	1	1							3
*B. rubigina* (Wang & Zhao)	CL, ZN		28	953	658	11	664	50	56	711		106				63	80	73	29
*B. syzygii* White & Tsuruta*	ZN		35	20	31	2	102	11	16			2							2
*B. tuberculata* (Bezzi)^1^	ME		1	1	10		4		3			13							4
*B. zonata* (Saunders)^1^	ME			2			1		1	2		48	63	2	69	11			1
*** Dacus ***																			
*D. ciliatus* Loew^3^																			
*D. longicornis* (Wiedemann)^3^	CL	3			1	5	12	9	25	16	1	71	10	1	23	7		1	40
*** Zeugodacus ***																			
*Z. apicalis* (de Meijere)*	CL								1										
*Z. atrifacies* (Perkins)*	CL						23												
*Z. caudatus* (Fabricius)^4^	CL							4			9	1		2	45	33			
*Z. cilifer* (Hendel)	CL							1				1							
*Z. cucurbitae* (Coquillett)^3^	CL, ZN	141	5	13	10	2	83	64	550	8	14	982	308	198	334	236			67
*Z. diaphorus* (Hendel)*^8^	CL													1	3	8			
*Z. diversus* (Coquillett)^3^	ME^6^											40							9
*Z. hochii* (Zia)^3^	CL^5^			1					1		9							2	
*Z. incisus* (Walker)*	CL			4	32		29	1				1				1			
*Z. infestus* (Enderlein)*	CL						1												
*Z. tau* (Walker)^3^	CL	273	434	30	11	10	33	69	383	15	115	672	143	191	82	169	1	1	103
*Z. madhupuri* Leblanc & Doorenweerd**	CL										4								
**NEUROPTERA: CHRYSOPIDAE**																			
*Ankylopteryx anomala* (Brauer)*	ME		5	14	13	251	53	88	1	2	5016	2					77	103	1

* New country record for Bangladesh. ** New species described in this paper. ^1^ Polyphagous fruit pest. ^2^ Oligophagous fruit pest. ^3^ Cucurbit fruit pest. ^4^ Cucurbit flower pest. ^5^ Zingerone attraction also known, but not recorded in Bangladesh. ^6^ Bangladesh specimens cited here collected by hand. ^7^ Previously reported as *Bactrocera* sp (possibly *B. bhutaniae*). ^8^ Previously reported as *Bactrocera* species 45 (likely *B. propinqua*). ^8^ Erroneously recorded as *Bactrocera bogorensis* in Leblanc et al. (2014).

### Morphological terms and taxonomic assignment

Morphological terminology used in the descriptions follows White (1999) and assignment of species to genera follows Doorenweerd et al. (2018). The genus *Zeugodacus*, of which a new species is described in this paper, is treated as separate from *Bactrocera* Macquart and *Dacus* Fabricius, based on recent molecular-based phylogenetic assessments (Krosch et al. 2012; Virgilio et al. 2015; Dupuis et al. 2017; San Jose et al. 2018a). Despite recent efforts to reassign species to subgenera (e.g., Hancock and Drew 2018 a, b), the understanding of higher relationships of species within Dacini is still in state of flux, and a number of traditionally recognized subgenera and species complexes (Drew and Romig 2013) are demonstrated to be polyphyletic groups of convenience defined on the basis of highly homoplastic morphological characters and male lure relations (e.g., Leblanc et al. 2015b; San Jose et al. 2018a; Catullo et al. 2019). For this reason, we have not attempted to include subgenera in the country’s species list.

### DNA extraction, PCR and sequencing

Methods for DNA extraction, PCR primers and conditions, and Sanger sequencing follow those of San Jose et al. (2018a). We attempted to amplify and sequence regions of the Cytochrome C Oxidase I (COI) and Elongation Factor 1-alpha (EF1-alpha) genes. It has previously been shown that COI cannot be used to differentiate *Bactrocera dorsalis* from *B. carambolae* (San Jose et al. 2018b). However, we found that there are five diagnostic single nucleotide polymorphisms (SNP’s) that separate *B. dorsalis* from *B. carambolae* in the 762 base-pair (bp) fragment of EF1-alpha that we used for multi-marker phylogenetic studies (San Jose et al. 2018a). We therefore sequenced this segment to confirm or refute the identity of *B. carambolae*. For *Zeugodacus madhupuri*, we attempted to amplify a large section of 1540 bp of COI as well as EF1-alpha, but we only successfully amplified the COI-3P region. Amplified regions of COI-5P proved to be nuclear pseudogenes after sequencing (data not shown), and EF1-alpha did not yield any PCR product, possibly due to degradation of the template DNA. We aligned newly generated sequences with the published data of EF1-alpha or COI, respectively, from San Jose et al. (2018a) and performed maximum likelihood analyses using IQTree (Nguyen et al. 2015). We allowed IQTree to determine the substitution model via its integrated modeltest and ran a standard maximum likelihood analyses with 1000 ultrafast bootstraps and 1000 Sh-aLRT bootstraps. We consider branches with support values >95% for ultrafast bootstraps and >80% for Sh-aLRT bootstraps as well supported. Resulting trees were optimized for publication using FigTree 1.4.3 and Adobe Illustrator. Data from this study are available from the BOLDSYSTEMS Digital Repository:

https://doi.org/10.5883/DS-BANG01.

### Estimating biodiversity

We used EstimateS software (Colwell 2013) to generate species accumulation curves. We estimated species diversity with the incidence-based Chao 2 algorithm, which does not include abundance in its extrapolation, thereby avoiding abundance bias in our data related to how strongly each species is attracted the lures and controlling for the predominance of a few agricultural pests in the samples. Diversity estimations were done comparing forest and rural sites, and the individual protected forest areas separately, with 100 randomizations without replacement for confidence intervals. It is understood that diversity estimates are underestimations, because they are based solely on species attracted to the male lures used in our sampling.

### Abbreviations

**AERE** Atomic Energy Research Establishment, Dhaka, Bangladesh

**UHIM** University of Hawaii Insect Museum, Honolulu, HI, USA

**USDA** United States Department of Agriculture

**WFBM** William F. Barr Entomological Museum, University of Idaho, Moscow, ID, USA

## Results

Between April 2013 and September 2018, we collected a total of 23,939 specimens of Dacine fruit flies, representing 29 species (Table 1), among 1012 samples (372 cue-lure, 357 methyl eugenol, 271 zingerone; and a few others hand-collected or bred from fruit) across 383 sites (Fig. 2). We report a number of new country occurrence records, including a major pest species in need of management attention (*Bactrocera carambolae*) and describe a new species, increasing the number of species of Tephritidae in Bangladesh from 27 (Leblanc et al. 2013, 2014; Hossain and Khan 2013; Khan et al. 2015, 2017) to 37 (29 Dacini and eight from other tribes).

### Biodiversity and species accumulation curves

**Figure 3.**
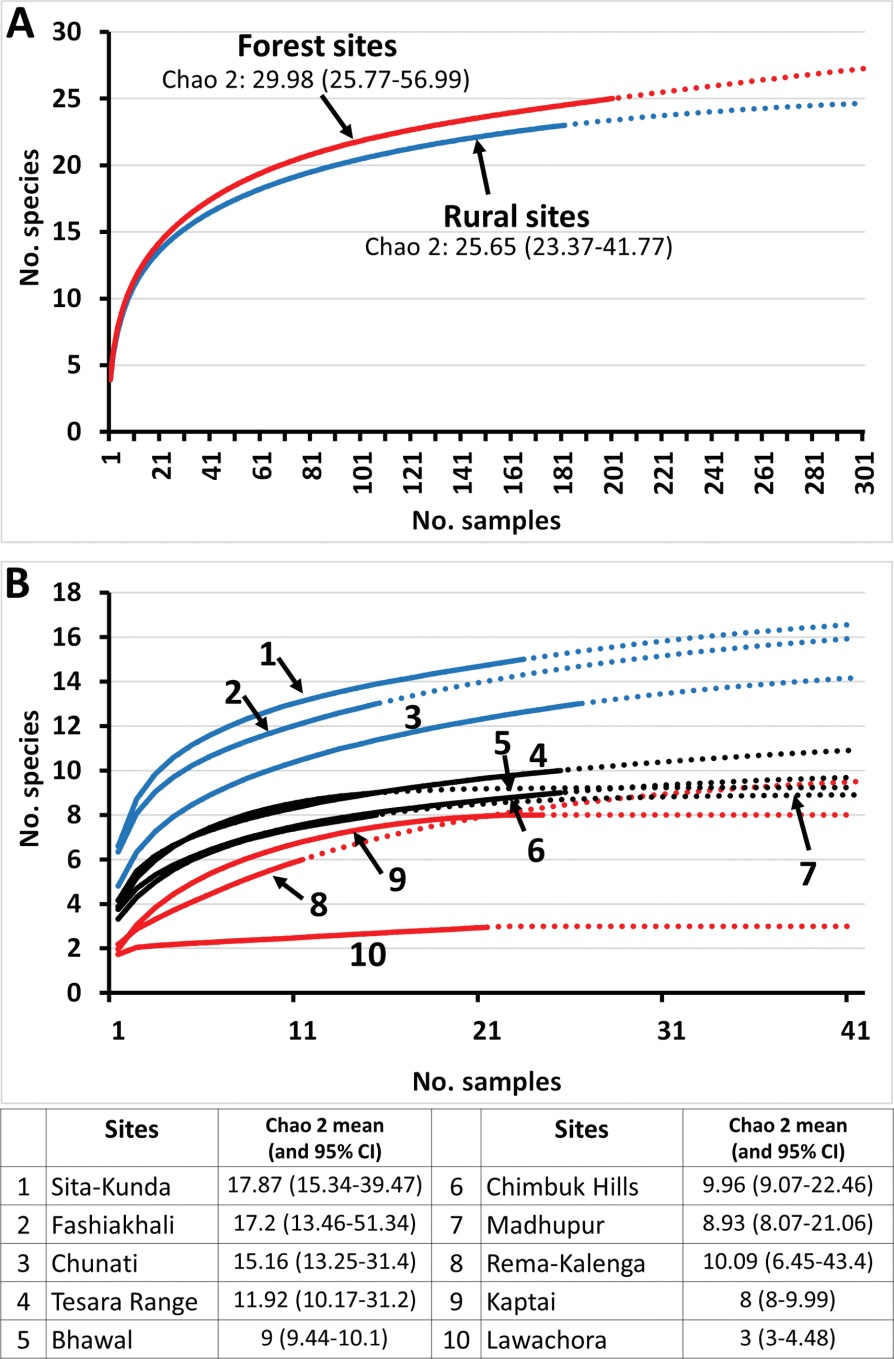
Species accumulation curves for species collected in the rural and forest sites through the whole sampling period (2013–2018) (**A**) and for the individually surveyed protected forest areas (**B**). Estimates of species numbers based on the Chao 2 estimator, with the 95% confidence interval ranges). Data used to generate these curves and estimates include two species not yet definitely identified and not included on Table 1.

Rural sites were dominated by three pest species: *Bactrocera dorsalis* (61.3% of specimens captured), *Zeugodacus cucurbitae* (Coquillett) (17.5%), and *Z. tau* (Walker) (12.6%). Forest sites also yielded large numbers of *B. dorsalis* (39.1%), as well as the non-economically important *B. rubigina* (Wang & Zhao) (41.4%), whereas cucurbit pests were less common (2.6% *Z. cucurbitae* and 9.2% *Z. tau*). The Chao 2 algorithm estimated overall number of species is 30.0 in forest sites and 25.7 in rural sites (Fig. 3A). Among the surveyed protected forests (Fig. 3B), the highest diversity was collected in three locations in the Chattogram District: Sita-Kunda Eco-Park, (15 species), Fashiakali Wildlife Sanctuary (12 species) and Chunati Wildlife Sanctuary (12 species), with the estimated Chao 2 number of species ranging from 15 to 18. Most other sites had a moderate diversity of 6–10 species, with estimated numbers of 8–12 species. For unclear reasons, only three species were collected in Lawachora National Park, possibly due to trapping done during the tail end of the rainy season (October 2016) and/or its relatively small size and geographic isolation from other forested areas within a densely populated environment dominated by agriculture. Paradoxically, the smallest sampled protected area, Sita-Kunda Eco-Park (808 ha) yielded the highest species diversity. The estimated total number of species in Bangladesh, based on the Chao 2 algorithm from a species accumulation curve including all sites, is 37.5 species, relatively few compared to the 118 species known to occur in the Indian subcontinent. A higher estimate might have been attained had species not attracted to lures been more actively collected and had access to the Chattogram Hills tracts forests not been severely restricted due to security concerns.

### *Bactrocera carambolae* new to Bangladesh

**Figure 4.**
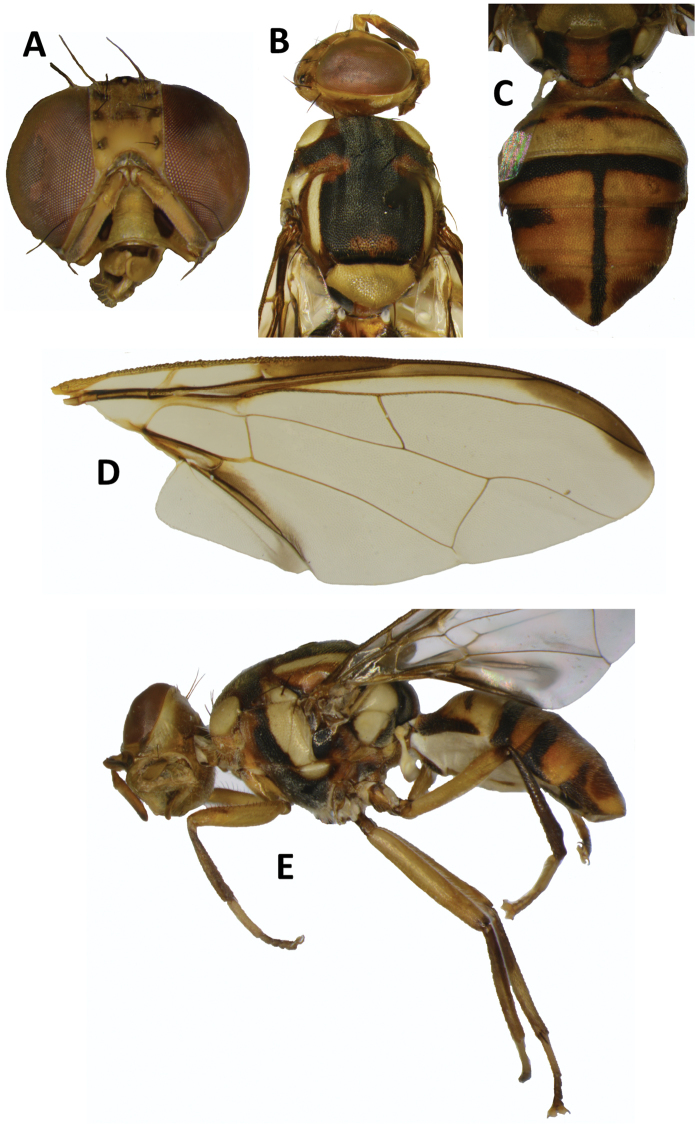
*Bactrocera carambolae* collected in Bangladesh **A** head **B** head and scutum **C** abdomen **D** wing **E** lateral view.

We collected 167 specimens of *B. carambolae* among 55 methyl eugenol samples, mostly in protected forest sites in the Chattogram District (Table 1, Fig. 2). One specimen morphologically consistent with *B. carambolae* was collected further north, in the Rema-Kalenga Wildlife Sanctuary (Sylhet Division) (UHIM molecular voucher ms07278), but its identity could not be confirmed molecularly because the amplification of EF1-alpha failed repeatedly, possibly due to degradation. All specimens are morphologically consistent with the diagnostic features of *B. carambolae*: subapical spots on fore femora, costal band slightly overlapping and expanded beyond apex of R_2+3_, presence of narrow transverse black band across anterior margin of tergum III, widening to cover lateral margins (Drew and Hancock 1994; Drew and Romig 2013) (Fig. 4). Species identity of a selection of nine specimens was further confirmed through sequencing of a region of EF1-alpha with five diagnostic SNP’s that differentiate *B. carambolae* from *B. dorsalis* (Genbank accession numbers MG683467, MG683640, MN413902–MN413909, MN418232–MN418240; Supplementary material 1: Figure S1). The discovery of *B. carambolae* in Bangladesh is a significant westward extension of the known distribution (Fig. 1) of this polyphagous fruit pest (>74 known fruit hosts in 26 families in Asia (Allwood et al. 1999)), until recently known to occur from southern portions of Vietnam, Thailand and Cambodia, Peninsular Malaysia, Java, Borneo, and south to Lombok (Indonesia), as well Andaman Island and South America (introduced in 1975). The origin of this pest species in forest habitats in south-eastern Bangladesh is enigmatic. It may reflect a relatively recent introduction, not detected during the 2013–2015 surveys which included agricultural environments. Alternatively, it may represent the extreme natural western range of its populations, possibly in expansion, if the species is demonstrated to be widespread across southern Myanmar. Recently, *B. carambolae* was demonstrated to occur in northwestern Thailand (Fig. 1) (San Jose et al. 2018b). Clearly, additional surveys to delimitate the range of this invasive pest in Bangladesh and Myanmar, focusing on trapping and host fruit surveys in agricultural environments, are of paramount priority.

#### Zeugodacus (Sinodacus) madhupuri

Taxon classificationAnimaliaDipteraTephritidae

Leblanc & Doorenweerd sp. nov.

33FAE2F4-FF38-58EA-A8F1-A490C3033751

http://zoobank.org/A992E0B1-F808-4744-9FE1-9ABD7F674FDF

Figs 6A–D ; 7A–C

##### Holotype.

Male. Labelled: “Bangladesh, Tangail District, Madhupur National Park, 24.702375N, 90.086325E, 5–13-ix-2018, M. Aftab Hossain, FFBn-316, cue-lure”, labelled as molecular voucher ms08804. Deposited at UHIM. **Paratypes**: One male. Labelled: “Bangladesh, Tangail District, Madhupur National Park, 24.704048N, 90.077770E, 5–13-ix-2018, M. Aftab Hossain, FFBn-311, cue-lure”. Deposited at WFBM. Two males labelled: “Bangladesh, Tangail District, Madhupur National Park, 24.703023N, 90.078774E, 5–13-ix-2018, M. Aftab Hossain, FFBn-312, cue-lure”, labelled as molecular vouchers ms08805 and ms08806. Deposited at UHIM.

##### Differential diagnosis.

*Zeugodacus madhupuri* is similar to the Indian Zeugodacus (Sinodacus) brevipunctatus (David & Hancock) (David et al. 2017), but differs in that the fuscous medial band and lateral markings on the abdomen are pale and less extensive than in *Z. brevipunctatus*, dark marking on legs are fulvous rather than fuscous, and *Z. madhupuri* consistently has two pairs of equally well-developed scutellar setae. *Zeugodacus brevipunctatus*, along with most other species of subgenus Sinodacus Zia has only one pair of scutellar setae (Hancock and Drew 2018a).

##### Molecular diagnostics.

**Figure 5.**
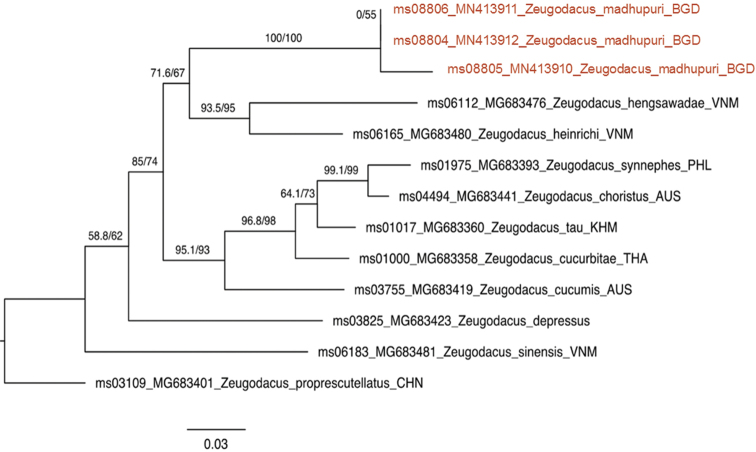
Maximum likelihood tree of COI-3P sequences of *Zeugodacus madhupuri* sp. nov. and its closest relatives in COI. Taxa names include UHIM ‘ms’ molecular voucher numbers, GenBank accessions and ISO three letter country codes. The record of *Zeugodacus hengsawadae* was published as Zeugodacus nr. tau in San Jose et al. (2018a). The scale bar indicates substitutions per site; values on the branches indicate ultrafast bootstrap support values and Sh-aLRT bootstrap values, respectively.

We obtained COI-3P sequences for three specimens, aligned them with the available COI-3P sequences from San Jose et al. (2018a) and performed maximum likelihood analyses. The full tree is available in Supplementary material 2: Figure S2, and a subset of *Z. madhupuri* and its closest relatives is shown in Figure 5. Based on our reference dataset, the new species is most similar to *Z. hengsawadae* (Drew & Romig) and *Z. heinrichi* (Hering) at around -11% pairwise distance and can be diagnosed reliably using COI. Note however that *Zeugodacus brevipunctatus* was not represented in our COI dataset.

##### Description of adult.

**Figure 6.**
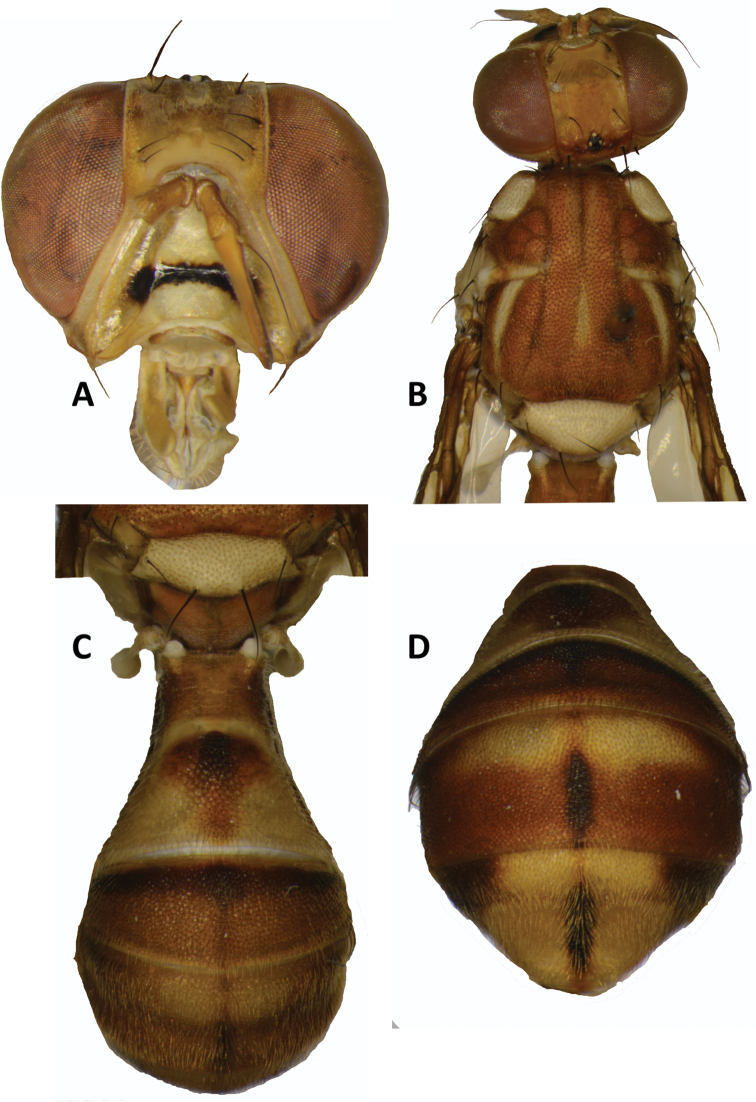
*Zeugodacus madhupuri***sp. nov.A** head **B** head and scutum **C–D** abdomen.

***Head*** (Fig. 6A, B). Vertical length 1.65–1.95 mm. Frons, of even width, 1.11–1.23 times as long as broad; fulvous with anteromedial hump covered by short red-brown hairs; orbital setae large-sized and dark fuscous and strong: one pair of superior and three pairs of inferior fronto-orbital setae present, the most anterior pairs nearly contiguous; lunule yellow. Ocellar triangle black. Vertex fuscous. Face fulvous to yellow with a broad transverse black band at mid height; length 0.58–0.75 mm. Genae fulvous, with or without a faint fuscous subocular spot; red-brown seta present. Occiput fulvous and yellow along eye margins; with two pairs of large occipital dorsal setae and lateral occipital rows with 5–8 light to dark setae. Antennae with scape, pedicel and first flagellomere fulvous and arista black (fulvous basally); length of segments: 0.20–0.25 mm; 0.25–0.35 mm; 0.83–0.93 mm.

***Thorax*** (Fig. 6B). Scutum fulvous with very narrow median and lateral faint fuscous longitudinal lines. Pleural areas fulvous except well-defined or faint red-brown area narrowly along anterior margin of mesopleural stripe, over most of anepimeron, and above mid coxae. Yellow markings as follows: postpronotal lobes (narrowly fulvous anteriorly); notopleura (notopleural callus); broad mesopleural (anepisternal) stripe, reaching level of anterior notopleural seta dorsally, continuing to katepisternum as a broad transverse spot, anterior margin straight or slightly convex; anatergite (with or without posterior margin narrowly red-brown); anterior 75% of katatergite (remainder fulvous); a narrow medial postsutural vitta and two narrow lateral postsutural vittae tapering posteriorly ending before or faintly reaching intra-alar setae, and prolonged narrowly anteriorly beyond notopleural suture. Postnotum fulvous. Scutellum yellow except for very narrow black basal band. Setae: four scutellar (both pairs well developed); prescutellar absent; one intraalar; one posterior supraalar; anterior supraalar absent; one mesopleural; two notopleural; four scapular; all setae well developed and red-brown. A weakly to well-developed postpronotal seta present in some specimens.

**Figure 7.**
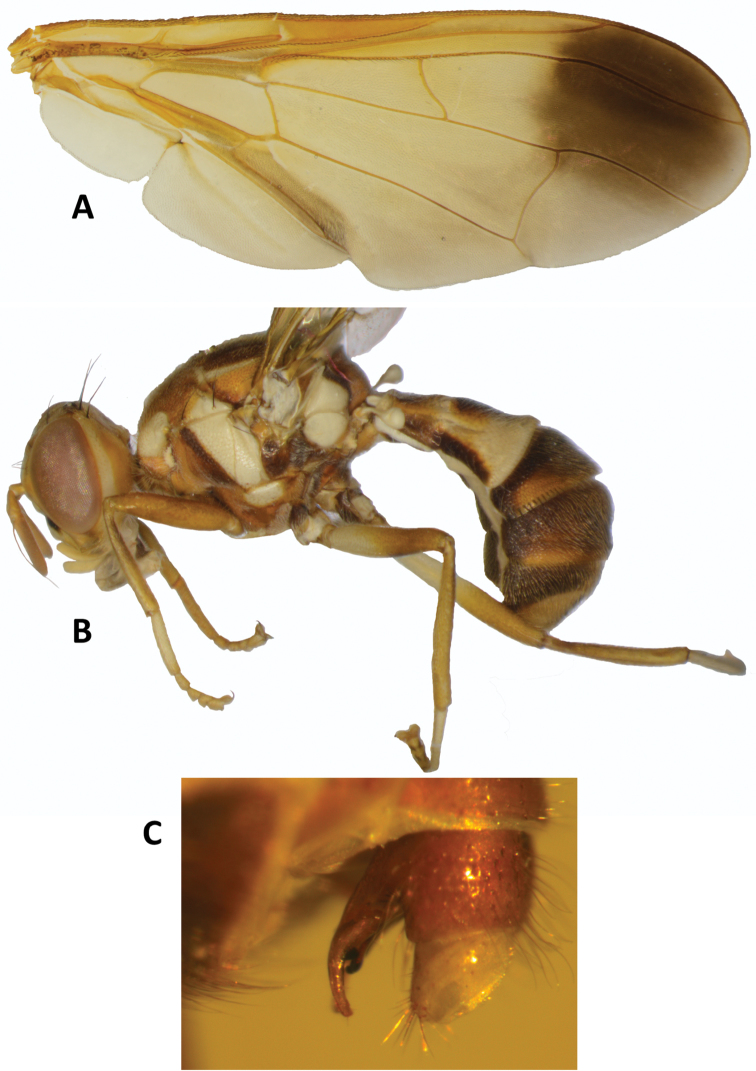
*Zeugodacus madhupuri* sp. nov. **A** wing **B** lateral view **C** terminal abdominal segment.

***Legs*** (Fig. 7B). Fore coxae basally and posteriorly dark fuscous and anteroapically fulvous. Mid and hind coxae predominantly dark fuscous. Trochanters fulvous. Femora fulvous, with basal half of mid femora and three-fifths of hind femora yellow; tibiae fulvous with apical black spur on mid tibiae; tarsi fulvous to yellow.

***Wings*** (Fig. 7A). Length 6.78–7.78 mm; basal costal (bc) and costal (c) cells fuscous; microtrichia in outer corner of cell costal only; remainder of wings with a pale fulvous tint except fuscous subcostal cell, broad fuscous costal band overlapping confluent with R_4+5_, a large dark fuscous apical spot from apex of R_2+3_, and englobing apical portions of veins R_4+5_ and M (from interception with dm-cu), a broad fuscous anal streak ending at apex of A_1_ + CuA_2_; dense aggregation of microtrichia around A_1_ + CuA_2_; supernumerary lobe well developed.

***Abdomen*** (Figs 6C, D, 7C). Elongate oval and petiolate; terga free; pecten present on tergum III; posterior lobe of surstylus long (Fig. 7C); abdominal sternum V with a shallow concavity on posterior margin. Tergum I as long as wide and sterna I and II longer than wide. Tergum I fulvous with apical margin narrowly yellow. Tergum II yellow with a median fulvous heart-shaped marking with an inner anteromedial fuscous marking. Terga III–V fulvous with fuscous along basal margin of tergum III, a narrow median longitudinal band reaching apex of tergum V, narrowly along lateral margins of terga III–IV, and as broad lateral markings on tergum V, anterior to ceromata, and with pale fulvous along base of tergum IV and on tergum V medially, anterior to ceromata (shining spots), which are also pale fulvous.

##### Etymology.

The species name is an adjective that refers to the Madhupur National Park, where all specimens were collected.

### Other new records

*Bactrocera abbreviata* (Hardy) new country record: One specimen collected in zingerone-baited trap, in October 2016, at the Institute of Nuclear Medicine and Allied Sciences (Chattogram). Known from the Philippines, China and Thailand (Drew and Romig 2013). Doorenweerd et al. (2018) noted that it may be conspecific with and junior synonym to *B. bipustulata* Bezzi, known from Sri Lanka and southern India. It has been bred in Thailand from *Chionanthus ramiflorus* and *Olea salicifolia* (family Oleaceae) (Allwood et al. 1999).

*Bactrocera pendleburyi* (Perkins) new country record: Eight specimens in six zingerone samples in forest sites of Chattogram District (Sita-Kunda, Chunati, Fashiakhali). Previously known from Peninsular Malaysia and Thailand, its presence in Bangladesh is a significant range extension. A non-pest species bred from *Symplocos cochinchinensis*, *S. racemosa* (Symplocaceae), and *Gmelina arborea* (Verbenaceae) (Allwood et al. 1999). A closely related species with entirely fulvous femora, *B. clarifemur* Leblanc & Doorenweerd, was recently described from Vietnam (Leblanc et al. 2018a). All Bangladesh specimens belong to *B. pendleburyi*.

*Bactrocera syzygii* White & Tsuruta new country record: Formerly known only from a small series of specimens bred from rose-apple (*Syzygium jambos*) in Sri Lanka (Drew and Romig 2013), it was recently recorded from India (David et al. 2017). The use of zingerone-baited traps collected large numbers in Sri Lanka (Leblanc et al. 2018b), Bangladesh (Table 1), and as far east as Vietnam (Leblanc et al. 2018a) and south as Sarawak and Sulawesi (CD, unpublished), consistent with the widespread distribution of rose-apple.

*Zeugodacus apicalis* (de Meijere) new country record: One specimen collected in cue-lure in Chattogram District (Rangunia Upazila) in November 2014. Widespread from China south to Sulawesi, it breeds on flowers of *Trichosanthes wawraei* (Cucurbitaceae) (Drew and Romig 2016). The Bangladesh new record is a significant westward extension.

*Zeugodacus atrifacies* (Perkins) new country record: A total of 23 specimens collected in 12 cue-lure samples, all in Chunati Wildlife Sanctuary. This species is widespread, from Bhutan east to Vietnam and south to Sarawak. This validates the record from India cited by Norrbom et al. (1999) that could not be confirmed by Drew and Romig (2013).

*Zeugodacus diaphorus* (Hendel) new country record and correction: 12 specimens in five cue-lure samples, all in rural sites. This species was erroneously identified as B. (Sinodacus) bogorensis (Hardy) in Leblanc et al. (2014), based on a comparison with specimens (of *Z. diaphorus*) in the UHIM, erroneously identified as *B. bogorensis* by Elmo Hardy. The record was published before keys to species (Drew and Romig 2016) became available and before we had access to a specimen of *Z. diaphorus* from Vietnam for molecular comparison. The record of *Z. diaphorus*, a species widespread from Sri Lanka and India East to Taiwan and South to Java is far more plausible than of *Z. bogorensis*, a species known only from Indonesia (Drew and Romig 2013). We therefore do not believe that *Z. bogorensis* is present in Bangladesh.

*Zeugodacus incisus* (Walker) new record: 67 specimens collected from 31 cue-lure samples, almost all in protected forest. Widespread from India to Vietnam and South to Peninsular Malaysia.

*Zeugodacus infestus* (Enderlein) new record: A single specimen collected at cue-lure in Sita-Kunda Eco-Park in July 2017. Significant range extension of a common species previously known from Vietnam, Thailand, Peninsular Malaysia Java, and Sumatra.

*Bactrocera bhutaniae* Drew & Romig confirmation of record: We confirm the identity of *Bactrocera* sp. (possibly *B. bhutaniae*) in Leblanc et al. (2014) as belonging to this species.

*Bactrocera propinqua* (Hardy & Adachi) confirmation of record: We confirm that the species previously reported as *Bactrocera* species 45 (likely *B. propinqua*) (Leblanc et al. 2013, 2014) as belonging to this species.

**Other Tephritidae**:

*Diarrhegma modestum* (Fabricius) (Acanthonevrini) was originally collected in Dhaka by Hossain and Khan (2013), and more recently in rural areas of Rajshahi District.

Khan et al. (2017) recorded the following bamboo-shoot fruit flies in Dhaka: *Felderimyia gombokensis* Hancock & Drew, *Rioxoptiolona dunlopi* (van der Wulp), and *R. vaga* (Wiedemann) (all Acanthonevrini), and *Acroceratitis distincta* (Zia), *A. ceratitina* (Bezzi) and *Gastrozona soror* (Schiner) (all Gastrozonini). *Gastrozona soror* was also collected by hand in rural areas of Feni District (Chattogram).

*Tephraciura basimacula* (Bezzi) (Tephrellini) new record: one specimen was hand-collected at the AERE, in Dhaka. This species, also known from southern India and Sri Lanka, breeds in flowerheads or seedpods of Acanthaceae (Hancock 2010).

### Non-target records

**Figure 8.**
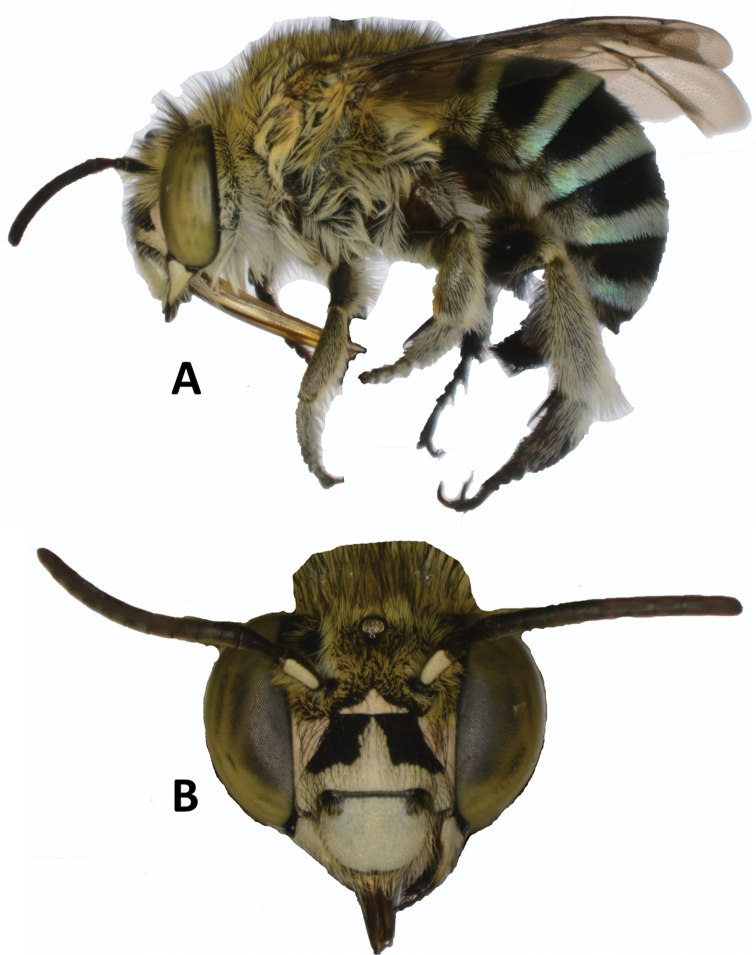
Amegilla (Zonamegilla) sp. (Hymenoptera: Apidae) collected in zingerone traps in Bangladesh. **A** Face **B** lateral view.

A total of 5626 specimens of *Ankylopteryx anomala* (Brauer) (Neuroptera, Chrysopidae) were collected in methyl eugenol traps, almost all in the forested areas. This species is widespread across tropical Asia, from Sri Lanka to Taiwan, and its attraction to methyl eugenol is well documented (Leblanc et al. 2015c). Two unidentified moths in the family Crambidae were caught in two separate methyl eugenol traps in Sita-Kunda Eco-Park. These may be real instances of attraction, because attraction of two species of flower-visiting crambids was demonstrated in Hawaii (Leblanc et al. 2009). Likewise, six specimens of one unidentified bee species in the genus Amegilla (subgenus Zonamegilla) (Hymenoptera, Apidae) (Fig. 8A, B) were collected in zingerone-baited traps in Sita-Kunda (three specimens in three traps), Chimbuk Hills (3 specimens in one trap) and Chunati (one specimen in one trap), and one specimen of the same species entered a methyl eugenol trap in Chattogram. The zingerone attraction record is credible and worth further investigation. One specimen of *Amegilla calceifera* (Cockerell) was also caught in a zingerone trap in Nepal in 2017 (LL, unpublished). The single specimen in the methyl eugenol trap may be accidental, though honeybee attraction to that lure has been reported (Leblanc et al. 2009).

### New records of fruit fly parasitoids

*Psyttalia fletcheri* (Silvestri) (Hymenoptera, Braconidae) new record: One kilogram of infested snake gourd yielded 427 fruit fly puparia, from which emerged 81 specimens of *Zeugodacus cucurbitae*, 286 specimens of *Z. tau*, and 43 specimens of *P. fletcheri*. A laboratory colony of this species was established at AERE in preparation for a pilot area-wide control program. Adult *P. fletcheri* are fed with a 10% sugar solution and honey, and oviposit in third instar larvae of *Z. cucurbitae* and *Z. tau*. *Psyttalia fletcheri* was rarely observed in commercially cultivated crop fields, likely due to the frequent applications of pesticides and low prevalence of alternate wild host fruits surrounding the fields.

*Spalangia* sp. and *Pachycrepoideus vindemmiae* (Rondani) (Hymenoptera, Pteromalidae) new records: Two 5 kg bottle gourds (*Lagenaria siceraria*) each infested with larvae of *Z. cucurbitae* and *Z. tau*, yielded 383 puparia and seven *Spalangia* and 387 pupae and nine *P. vindemmiae*, respectively. Laboratory colonies of both parasitoids were established at AERE. Hosts determined to be suitable for both species are puparia of *Z. cucurbitae*, *Z. tau*, *B. dorsalis*, and *B. zonata* with a preference for *Z. cucurbitae* by *Spalangia* and for *B. dorsalis* by *P. vindemmiae*.

## Supplementary Material

XML Treatment for Zeugodacus (Sinodacus) madhupuri

## Supplementary materials

Supplementary material 1 Figure S1 Data type: statistical data Explanation note: Maximum likelihood tree based on elongation factor 1 alpha sequences from (San Jose et al. 2018) with newly generated *Bactrocera carambolae* sequences added. https://binary.pensoft.net/file/341426This dataset is made available under the Open Database License (http://opendatacommons.org/licenses/odbl/1.0/). The Open Database License (ODbL) is a license agreement intended to allow users to freely share, modify, and use this Dataset while maintaining this same freedom for others, provided that the original source and author(s) are credited.Luc Leblanc, M. Aftab Hossain, Camiel Doorenweerd, Shakil Ahmed Khan, Mahfuza Momen, Michael San Jose, Daniel Rubinoff

Supplementary material 2 Figure S2 Data type: statistical data Explanation note: Maximum likelihood tree based on cytochrome c oxidase I sequences from (San Jose et al. 2018) with newly generated *Zeugodacus madhupuri* sequences added, taxa names colored orange. https://binary.pensoft.net/file/341427This dataset is made available under the Open Database License (http://opendatacommons.org/licenses/odbl/1.0/). The Open Database License (ODbL) is a license agreement intended to allow users to freely share, modify, and use this Dataset while maintaining this same freedom for others, provided that the original source and author(s) are credited.Luc Leblanc, M. Aftab Hossain, Camiel Doorenweerd, Shakil Ahmed Khan, Mahfuza Momen, Michael San Jose, Daniel Rubinoff
