# Supplementary figures and images for: Six years of fruit fly surveys in Bangladesh: a new species, 33 new country records and discovery of the highly invasive Bactrocera carambolae (Diptera, Tephritidae)
Source: Zookeys. 2019 Sep 25;876:87–109. doi: 10.3897/zookeys.876.38096 (PMC6775173; doi:10.3897/zookeys.876.38096)

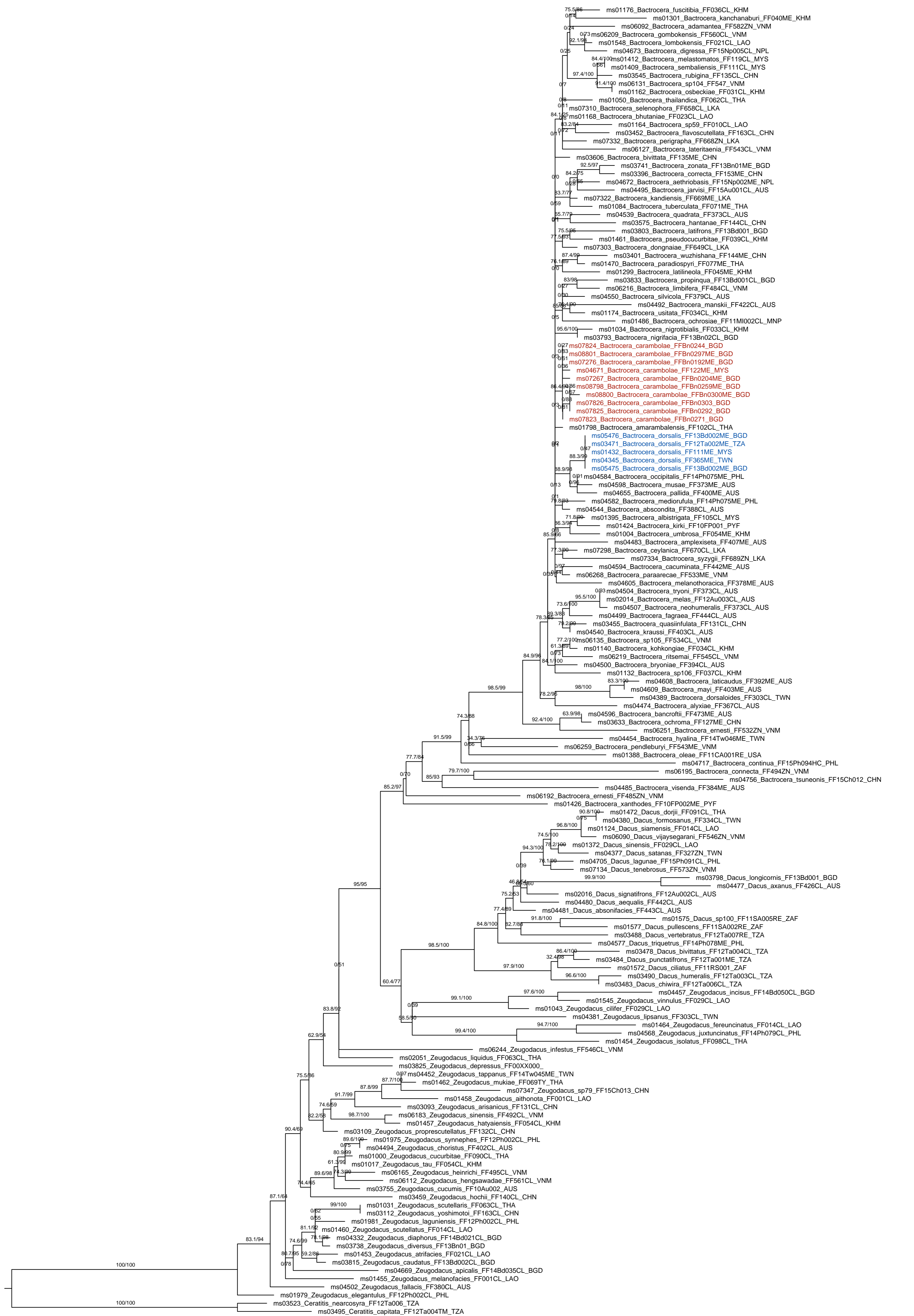

Supplement: Supplementary material 1 [file zookeys-876-087-s001.pdf]
