# Supplementary material for: Six years of fruit fly surveys in Bangladesh: a new species, 33 new country records and discovery of the highly invasive Bactrocera carambolae (Diptera, Tephritidae)
Source: Zookeys. 2019 Sep 25;876:87–109. doi: 10.3897/zookeys.876.38096 (PMC6775173; doi:10.3897/zookeys.876.38096)
Supplement: Supplementary material 2 [file zookeys-876-087-s002.pdf]

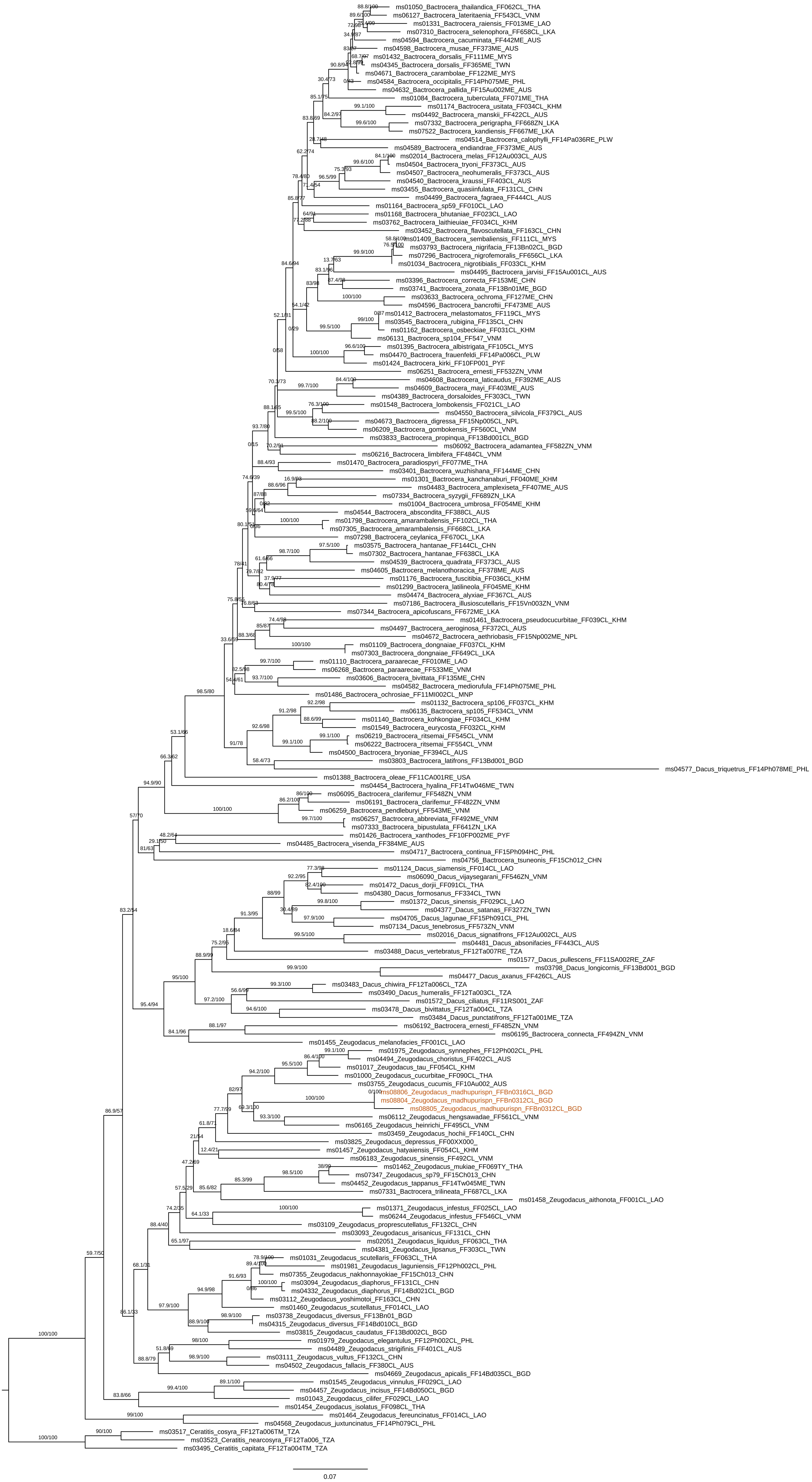
ms01050_Bactrocera_thailandica_FF062CL_THA
ms06127_Bactrocera_lateritaenia_FF543CL_VNM
ms01331_Bactrocera_raiensis_FF013ME_LAO
ms07310_Bactrocera_selenophora_FF658CL_LKA
ms04594_Bactrocera_cacuminata_FF442ME_AUS
ms04598_Bactrocera_musae_FF373ME_AUS
ms01432_Bactrocera_dorsalis_FF111ME_MYS
ms04345_Bactrocera_dorsalis_FF365ME_TWN
ms04671_Bactrocera_carambolae_FF122ME_MYS
ms04584_Bactrocera_occipitalis_FF14Ph075ME_PHL
ms04632_Bactrocera_pallida_FF15Au002ME_AUS
ms01084_Bactrocera_tuberculata_FF071ME_THA
ms01174_Bactrocera_usitata_FF034CL_KHM
ms04492_Bactrocera_manskii_FF422CL_AUS
ms07332_Bactrocera_perigrapha_FF668ZN_LKA
ms07522_Bactrocera_kandiensis_FF667ME_LKA
ms04514_Bactrocera_calophylli_FF14Pa036RE_PLW
ms04589_Bactrocera_endiandrae_FF373ME_AUS
ms02014_Bactrocera_melas_FF12Au003CL_AUS
ms04504_Bactrocera_tryoni_FF373CL_AUS
ms04507_Bactrocera_neohumeralis_FF373CL_AUS
ms04540_Bactrocera_kraussi_FF403CL_AUS
ms03455_Bactrocera_quasiinfulata_FF131CL_CHN
ms04499_Bactrocera_fagraea_FF444CL_AUS
ms01164_Bactrocera_sp59_FF010CL_LAO
ms01168_Bactrocera_bhutaniae_FF023CL_LAO
ms03762_Bactrocera_laithieuiae_FF034CL_KHM
ms03452_Bactrocera_flavoscutellata_FF163CL_CHN
ms01409_Bactrocera_sembaliensis_FF111CL_MYS
ms03793_Bactrocera_nigrifacia_FF13Bn02CL_BGD
ms07296_Bactrocera_nigrofemoralis_FF656CL_LKA
ms01034_Bactrocera_nigrotibialis_FF033CL_KHM
ms04495_Bactrocera_jarvisi_FF15Au001CL_AUS
ms03396_Bactrocera_correcta_FF153ME_CHN
ms03741_Bactrocera_zonata_FF13Bn01ME_BGD
ms03633_Bactrocera_ochroma_FF127ME_CHN
ms04596_Bactrocera_bancroftii_FF473ME_AUS
ms01412_Bactrocera_melastomatos_FF119CL_MYS
ms03545_Bactrocera_rubigina_FF135CL_CHN
ms01162_Bactrocera_osbeckiae_FF031CL_KHM
ms06131_Bactrocera_sp104_FF547_VNM
ms01395_Bactrocera_albistrigata_FF105CL_MYS
ms04470_Bactrocera_frauenfeldi_FF14Pa006CL_PLW
ms01424_Bactrocera_kirki_FF10FP001_PYF
ms06251_Bactrocera_ernesti_FF532ZN_VNM
ms04608_Bactrocera_laticaudus_FF392ME_AUS
ms04609_Bactrocera_mayi_FF403ME_AUS
ms04389_Bactrocera_dorsaloides_FF303CL_TWN
ms01548_Bactrocera_lombokensis_FF021CL_LAO
ms04550_Bactrocera_silvicola_FF379CL_AUS
ms04673_Bactrocera_digressa_FF15Np005CL_NPL
ms06209_Bactrocera_gombokensis_FF560CL_VNM
ms03833_Bactrocera_propinqua_FF13Bd001CL_BGD
ms06092_Bactrocera_adamantea_FF582ZN_VNM
ms06216_Bactrocera_limbifera_FF484CL_VNM
ms01470_Bactrocera_paradiospyri_FF077ME_THA
ms03401_Bactrocera_wuzhishana_FF144ME_CHN
ms01301_Bactrocera_kanchanaburi_FF040ME_KHM
ms04483_Bactrocera_amplexiseta_FF407ME_AUS
ms07334_Bactrocera_syzygii_FF689ZN_LKA
ms01004_Bactrocera_umbrosa_FF054ME_KHM
ms04544_Bactrocera_abscondita_FF388CL_AUS
ms01798_Bactrocera_amarambalensis_FF102CL_THA
ms07305_Bactrocera_amarambalensis_FF668CL_LKA
ms07298_Bactrocera_ceylanica_FF670CL_LKA
ms03575_Bactrocera_hantanae_FF144CL_CHN
ms07302_Bactrocera_hantanae_FF638CL_LKA
ms04539_Bactrocera_quadrata_FF373CL_AUS
ms04605_Bactrocera_melanothoracica_FF378ME_AUS
ms01176_Bactrocera_fuscitibia_FF036CL_KHM
ms01299_Bactrocera_latilineola_FF045ME_KHM
ms04474_Bactrocera_alyxiae_FF367CL_AUS
ms07186_Bactrocera_illusioscutellaris_FF15Vn003ZN_VNM
ms07344_Bactrocera_apicofuscans_FF672ME_LKA
ms01461_Bactrocera_pseudocucurbitae_FF039CL_KHM
ms04497_Bactrocera_aeroginosa_FF372CL_AUS
ms04672_Bactrocera_aethriobasis_FF15Np002ME_NPL
ms01109_Bactrocera_dongnaiae_FF037CL_KHM
ms07303_Bactrocera_dongnaiae_FF649CL_LKA
ms01110_Bactrocera_paraarecae_FF010ME_LAO
ms06268_Bactrocera_paraarecae_FF533ME_VNM
ms03606_Bactrocera_bivittata_FF135ME_CHN
ms04582_Bactrocera_mediorufula_FF14Ph075ME_PHL
ms01486_Bactrocera_ochrosiae_FF11MI002CL_MNP
ms01132_Bactrocera_sp106_FF037CL_KHM
ms06135_Bactrocera_sp105_FF534CL_VNM
ms01140_Bactrocera_kohkongiae_FF034CL_KHM
ms01549_Bactrocera_eurycosta_FF032CL_KHM
ms06219_Bactrocera_ritsemai_FF545CL_VNM
ms06222_Bactrocera_ritsemai_FF554CL_VNM
ms04500_Bactrocera_bryoniae_FF394CL_AUS
ms03803_Bactrocera_latifrons_FF13Bd001_BGD
ms04577_Dacus_triquetrus_FF14Ph078ME_PHL
ms01388_Bactrocera_oleae_FF11CA001RE_USA
ms04454_Bactrocera_hyalina_FF14Tw046ME_TWN
ms06095_Bactrocera_clarifemur_FF548ZN_VNM
ms06191_Bactrocera_clarifemur_FF482ZN_VNM
ms06259_Bactrocera_pendleburyi_FF543ME_VNM
ms06257_Bactrocera_abbreviata_FF492ME_VNM
ms07333_Bactrocera_bipustulata_FF641ZN_LKA
ms01426_Bactrocera_xanthodes_FF10FP002ME_PYF
ms04485_Bactrocera_visenda_FF384ME_AUS
ms04717_Bactrocera_continua_FF15Ph094HC_PHL
ms04756_Bactrocera_tsuneonis_FF15Ch012_CHN
ms01124_Dacus_siamensis_FF014CL_LAO
ms06090_Dacus_vijaysegarani_FF546ZN_VNM
ms01472_Dacus_dorjii_FF091CL_THA
ms04380_Dacus_formosanus_FF334CL_TWN
ms01372_Dacus_sinensis_FF029CL_LAO
ms04377_Dacus_satanas_FF327ZN_TWN
ms04705_Dacus_lagunae_FF15Ph091CL_PHL
ms07134_Dacus_tenebrosus_FF573ZN_VNM
ms02016_Dacus_signatifrons_FF12Au002CL_AUS
ms04481_Dacus_absonifacies_FF443CL_AUS
ms03488_Dacus_vertebratus_FF12Ta007RE_TZA
ms01577_Dacus_pullescens_FF11SA002RE_ZAF
ms03798_Dacus_longicornis_FF13Bd001_BGD
ms04477_Dacus_axanus_FF426CL_AUS
ms03483_Dacus_chiwira_FF12Ta006CL_TZA
ms03490_Dacus_humeralis_FF12Ta003CL_TZA
ms01572_Dacus_ciliatus_FF11RS001_ZAF
ms03478_Dacus_bivittatus_FF12Ta004CL_TZA
ms03484_Dacus_punctatifrons_FF12Ta001ME_TZA
ms06192_Bactrocera_ernesti_FF485ZN_VNM
ms06195_Bactrocera_connecta_FF494ZN_VNM
ms01455_Zeugodacus_melanofacies_FF001CL_LAO
ms01975_Zeugodacus_synnephes_FF12Ph002CL_PHL
ms04494_Zeugodacus_choristus_FF402CL_AUS
ms01017_Zeugodacus_tau_FF054CL_KHM
ms01000_Zeugodacus_cucurbitae_FF090CL_THA
ms03755_Zeugodacus_cucumis_FF10Au002_AUS
ms08806_Zeugodacus_madhupurispn_FFBn0316CL_BGD
ms08804_Zeugodacus_madhupurispn_FFBn0312CL_BGD
ms08805_Zeugodacus_madhupurispn_FFBn0312CL_BGD
ms06112_Zeugodacus_hengsawadae_FF561CL_VNM
ms06165_Zeugodacus_heinrichi_FF495CL_VNM
ms03459_Zeugodacus_hochii_FF140CL_CHN
ms03825_Zeugodacus_depressus_FF00XX000_
ms01457_Zeugodacus_hatyaiensis_FF054CL_KHM
ms06183_Zeugodacus_sinensis_FF492CL_VNM
ms01462_Zeugodacus_mukiae_FF069TY_THA
ms07347_Zeugodacus_sp79_FF15Ch013_CHN
ms04452_Zeugodacus_tappanus_FF14Tw045ME_TWN
ms07331_Bactrocera_trilineata_FF687CL_LKA
ms01458_Zeugodacus_aithonota_FF001CL_LAO
ms01371_Zeugodacus_infestus_FF025CL_LAO
ms06244_Zeugodacus_infestus_FF546CL_VNM
ms03109_Zeugodacus_proprescutellatus_FF132CL_CHN
ms03093_Zeugodacus_arisanicus_FF131CL_CHN
ms02051_Zeugodacus_liquidus_FF063CL_THA
ms04381_Zeugodacus_lipsanus_FF303CL_TWN
ms01031_Zeugodacus_scutellaris_FF063CL_THA
ms01981_Zeugodacus_laguniensis_FF12Ph002CL_PHL
ms07355_Zeugodacus_nakhonnayokiae_FF15Ch013_CHN
ms03094_Zeugodacus_diaphorus_FF131CL_CHN
ms04332_Zeugodacus_diaphorus_FF14Bd021CL_BGD
ms03112_Zeugodacus_yoshimotoi_FF163CL_CHN
ms01460_Zeugodacus_scutellatus_FF014CL_LAO
ms03738_Zeugodacus_diversus_FF13Bn01_BGD
ms04315_Zeugodacus_diversus_FF14Bd010CL_BGD
ms03815_Zeugodacus_caudatus_FF13Bd002CL_BGD
ms01979_Zeugodacus_elegantulus_FF12Ph002CL_PHL
ms04489_Zeugodacus_strigifinis_FF401CL_AUS
ms03111_Zeugodacus_vultus_FF132CL_CHN
ms04502_Zeugodacus_fallacis_FF380CL_AUS
ms04669_Zeugodacus_apicalis_FF14Bd035CL_BGD
ms01545_Zeugodacus_vinnulus_FF029CL_LAO
ms04457_Zeugodacus_incisus_FF14Bd050CL_BGD
ms01043_Zeugodacus_cilifer_FF029CL_LAO
ms01454_Zeugodacus_isolatus_FF098CL_THA
ms01464_Zeugodacus_fereuncinatus_FF014CL_LAO
ms04568_Zeugodacus_juxtuncinatus_FF14Ph079CL_PHL
ms03517_Ceratitis_cosyra_FF12Ta006TM_TZA
ms03523_Ceratitis_nearcosyra_FF12Ta006_TZA
ms03495_Ceratitis_capitata_FF12Ta004TM_TZA
0.07
